# Case 3 / 2018 - Corrected Transposition of the Great Arteries with Natural Progression to Severe Biventricular Dysfunction and No Associated Defects in a 51-Year Old Man

**DOI:** 10.5935/abc.20180080

**Published:** 2018-05

**Authors:** Edmar Atik, Fidel Leal, Ivanhoé S. L. Leite

**Affiliations:** Instituto do Coração do Hospital das Clínicas da Faculdade de Medicina da Universidade de São Paulo, São Paulo, SP - Brazil

**Keywords:** Congenitally Corrected Transposition of Great Arteries, Ventricular Dysfunction / surgery, Heart Failure, Cardiac Output, Low, Syncope

## Clinical data

Dyspnea on exertion for two years, progressing to low cardiac output and syncope lately, treated with dobutamine and usual drugs for congestive heart disease (currently using furosemide 40 mg, spironolactone 25 mg and losartan 12.5 mg).

Physical examination: Good general condition, eupneic, acyanotic, normal pulse rate in the four limbs. Weight: 70 Kg, Height: 160 cm, blood pressure (right arm): 90/60 mmHg, HR: 94 bpm.

Precordium: Apex beat was not palpable, without systolic impulses. Low heart sounds, and low intensity heart murmur heard in the left lower sternal border. Liver was not palpable and lungs were clear.

## Complementary tests

**Figure 1:**
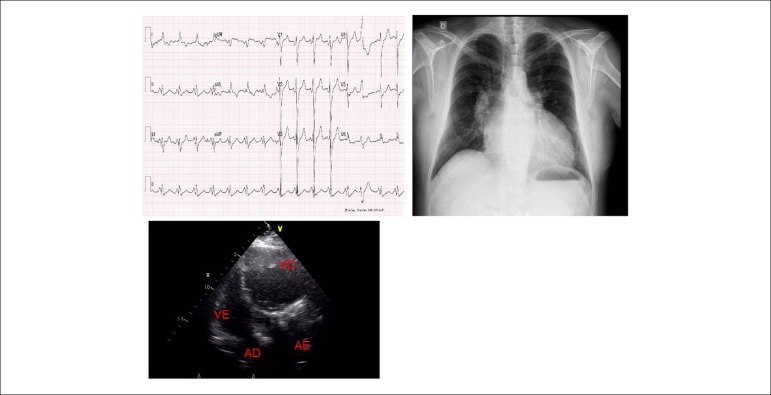
Electrocardiogram showing conduction abnormality in the left branch the, biatrial overload and T-wave orientation towards the left ventricle at right. Chest X-ray showing cardiomegaly with enlarged ventricle and left atrium. Four-chamber echocardiographic view showing enlarged right ventricle at left and deviation of the interventricular septum at right and enlarged left atrium.

**Electrocardiography**: Sinus rhythm, conduction abnormality seen in the left branch with long QRS duration (169 ms; AQRS = 0º), negative T-wave in I, aVL and V6 (AT = +155º), biatrial overload, and enlarged, peaked p-wave (AP+77º). (Figure 1).

**Chest radiography:**Enlarged heart due to round-shaped left ventricular arch and double-density left atrium with elevation of left bronchus. Congestion of pulmonary vessels, enlarged descending aorta, dilation of mid-aortic arch. Cardiothoracic index 0.61 (Figure 1).

**Echocardiography**: Atrioventricular and ventriculoarterial discordance, intact atrioventricular conduction. Ventricular septum is bulging to the right. Marked tricuspid insufficiency to the left (tricuspid annulus = 36 mm) and dilated atriums. Systolic dysfunction and diffuse hypokinesis of hypokinesis of right ventricle, TAPSE = 0.7 CM. Significant left ventricular dysfunction (Figure 1).

**Figure 2:**
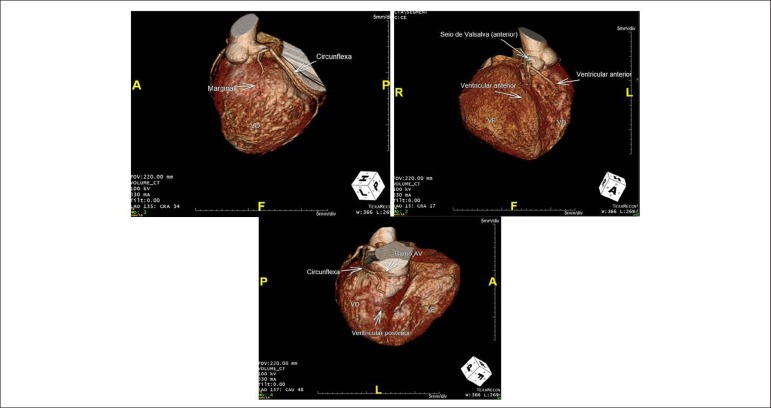
Computed tomography angiography of coronary arteries showing the right and left anterior ventricular branch originating from the anterior sinus of Valsalva (B). Larger arteries, composed by the circumflex, posterior ventricular and marginal arteries, arise from the posterior sinus of Valsalva that perfuses the entire right ventricle at left (A and C).

**Computed tomography coronary angiography**: Left-dominant coronary circulation. The right ventricle was perfused by an arterial branch originating at the posterior sinus of Valsalva and bifurcating into posterior circumflex artery, ventricular artery and marginal artery. It was also perfused by the anterior ventricular artery, a coronary artery branch that arises from the anterior Valsalva sinus. Left ventricle was perfused at right by the artery that arises from anterior Valsalva sinus as a thin branch and travels towards the anterior surface (Figure 2).

**Holter**: Sinus rhythm, with no arrhythmias.

**Myocardial magnetic resonance imaging**: Significant right and left ventricular dysfunction (EF = 29%; right ventricular end-systolic volume, RVESV = 154 mL/m^2^ and EF = 36%; RVESV = 73 mL/m^2^, respectively). Preserved right atrial function and enlarged left atrium. Delayed-enhancement in anterior and lower junction and in both ventricular outflow tracts. Significant tricuspid regurgitation.

**Ergospirometry**: Peak oxygen consumption of 16.4 ml/kg/min, 76% of peak VO2 (56% of peak VO2 predicted for age); respiratory compensation point was not reached. Slope VE/VCO2 of 31.

**Clinical diagnosis:** Corrected transposition of the great arteries with severe biventricular dysfunction and no associated defects.

**Clinical reasoning:** There were clinical evidence of corrected transposition of the great arteries, particularly a late ventricular dysfunction detected few years ago due to tiredness. This is corroborated by electrocardiographic signs, especially the orientation of ventricular repolarization characterized by orientation of the T-wave to the right. The diagnosis was well established by echocardiography and magnetic resonance imaging. The late ventricular dysfunction was probably caused by relative coronary insufficiency caused by systemic right ventricular hypertrophy, despite good irrigation seen in computed tomography coronary angiography.

**Differential diagnosis**: In adult patients, all other causes of ventricular dysfunction may be considered, including ischemic cardiomyopathy and dilated cardiomyopathy of other causes.

**Management**: Heart transplantation was indicated due to significant biventricular dysfunction.

**Comments**: Corrected transposition of the great arteries with no associated defects has an incidence of 10-15%. Both patients with natural progression of the disease and those who undergo surgical techniques for functional correction progress to different degrees of systemic right ventricular dysfunction in adult age.^1^^,^^2^ It becomes even worse with advanced age and occurs in 50-80% of these cases. From eight more advanced age cases reported in the literature, five of them had congestive heart failure.^3^ The congestive syndrome may be explained by relative coronary insufficiency related to the hypertrophied systemic right ventricle. In this regard, the decreased coronary flow has been well documented in the literature and recognized as a consequence of right ventricular dysfunction, and the main long-term sequela of this condition. Decreased coronary flow after vasodilation with adenosine, resulting in altered vasoreactivity and possible microcirculation was previously reported,^4^ which may explain the ventricular dysfunction. Therefore, the best option for these patients may be atrial and arterial anatomic repair by double switch operation in some stage of the disease.^1^^,^^2^
